# Identification of Potential Peptide Marker(s) for Evaluating Pork Meat Freshness via Mass Spectrometry-Based Peptidomics during Storage under Different Temperatures

**DOI:** 10.3390/foods11081144

**Published:** 2022-04-15

**Authors:** Zhenqian Wei, Chen Dai, Anthony P. Bassey, Changbo Tang, Yu Han, Chong Wang, Guanghong Zhou

**Affiliations:** 1 Key Laboratory of Meat Products Processing, College of Food Science and Technology, Nanjing Agricultural University, Ministry of Agriculture, Jiangsu Collaborative Innovation Center of Meat Production and Processing, Quality and Safety Control, Nanjing 210095, China; 2019108080@njau.edu.cn (Z.W.); bassey_ap44@outlook.com (A.P.B.); tangcb@njau.edu.cn (C.T.); 2020108026@njau.edu.cn (Y.H.); guanghong.zhou@hotmail.com (G.Z.); 2 Experimental Teaching Center of Life Science, Nanjing Agricultural University, Nanjing 210095, China; daichencpu@njau.edu.cn; 3 Laboratory of Genomics and Molecular Biomedicine, Department of Biology, University of Copenhagen, 2100 Copenhagen, Denmark

**Keywords:** pork meat, freshness, biomarker, peptidomics, peptide

## Abstract

This study applied peptidomics to investigate potential biomarkers for evaluating pork-meat freshness. The spoilage time points of pork meat stored at −2, 4, 10, and 25 °C were defined by evaluating meat freshness indicators (color, total viable count, pH, and total volatile basic nitrogen). Peptide MVHMASKE was identified as a potential peptide marker via multivariate analysis. Pearson correlation revealed a negative correlation between intensity of MVHMASKE and total viable count/total volatile basic nitrogen. In addition, the correlation between peptide content and the change in pork-meat freshness was verified using real-life samples, and the content of MVHMASKE showed a significant decline during storage under 4 and 25 °C, correspondingly reflecting the change of pork meat from fresh to spoiled. This study provides favorable evidence to evaluate pork-meat freshness by monitoring the change of peptide MVHMASKE in content based on mass spectrometry-based peptidomics.

## 1. Introduction

Pork is a protein-rich food for humans and one of the most highly consumed meats in China [1]. However, owing to its rich nutritional composition, pork meat is highly susceptible to deterioration largely due to microbiological metabolism regardless of the retail or storage conditions. A deterioration in freshness is accompanied by physicochemical changes, including off-odors, discoloration, and the formation of slime [1,2]. This leads to economic losses and is associated with food-safety issues and health risks owing to the harmful substances produced by microorganisms [3]. Therefore, the determination of meat freshness and improvements in food safety are crucial to ensure the quality of pork products and to ensure human safety.

Conventional evaluations of meat freshness include sensory assessments, analysis of physicochemical parameters, and microbiological monitoring [4,5]. Sensory evaluation, although simple and fast, is highly subjective, and can be influenced by the sensory acuity of the panelists, leading to inconsistencies that may require further laboratory evaluation [6]. Chemical and microbiological tests, such as total volatile basic nitrogen (TVB-N) and total viable count (TVC), can provide relatively precise data; however, they require a specialized experimental environment and involve time-consuming processes [6]. Other approaches, such as electrochemistry, Raman spectroscopy, and hyperspectral systems, have been applied to evaluate meat spoilage [7]. Nowadays, foodomics presents a new insight in evaluating meat freshness, especially the achievements in the exploration of seafood freshness [8]. Aru et al. [9] found that the metabolites, such as acetate, lactate, succinate, alanine, and branched chain amino acids, could be used as potential biomarkers for evaluating bivalve mollusk *Mytilus galloprovincialis* spoilage by a nuclear magnetic resonance (NMR) metabolomics approach. Li et al. [10] used proteomics method to screen out seven proteins (phosphoglucomutase-1, pyruvate kinase, kinesin-1 heavy chain, Troponin T, desmin, and actin) from the turbot (*Scophthalmus maximus*) muscle during post-mortem storage, and the abundance changes of those proteins were significantly correlated with TVB-N, K value, and TVC.

Several studies have reported that meat spoilage is directly related to the activity of microorganisms and endogenous enzymes on the metabolism of meat proteins [11]. Therefore, the use of proteins and their metabolites represents a supporting approach for evaluating meat freshness. Peptidomics, an emerging branch of proteomics [12], is a powerful tool in investigating meat science. Stachniuk et al. [13] identified guinea fowl specific peptide, LSADTEVVCGAPAIYLDFAR, as a reliable and stable biomarker for distinguishing guinea fowl meat from commonly consumed poultry species via a liquid chromatography coupled to a quadrupole-time of flight (Q-TOF) mass spectrometer (MS) approach. Kominami et al. [14] used label-free peptidomics to investigate proteolysis in a beef short plate during thaw-aging and identified degraded proteins and their cleavage sites. To date, peptidomics has been used to identify bioactive peptides [15,16], characterize proteolysis during food processing [17], and authenticate processed meat products [18].

The application of peptidomics to identify biomarkers for assessing meat quality has increased. Gallego et al. [19] reported that peptides APAPAPAPPKEEKI and PAPAPAPAPAPAPAPPKE identified at 9 months of curing could be potential markers for controlling curing time and final quality of dry-cured hams. Similarly, peptide biomarkers to assess the quality of aquatic products have been determined. Notably, Chen et al. [20] identified four endogenous peptide markers by UHPLC-Q-TOF that changed markedly with storage time, indicating their potential as shelf-life indicators for *Crassostrea (C.) gigas* during anhydrous preservation. To our knowledge, information on changes in endogenous peptides during pork preservation and their potential as meat-freshness biomarkers is limited.

Thus, in the present study, peptidomics was applied to provide an evaluation of pork-meat freshness under different storage conditions (−2 °C, controlled freezing point; 4 °C, refrigerated; 10 °C, retail storage; 25 °C, room temperature). UHPLC-LTQ-Orbitrap mass spectrometry combined with orthogonal partial least-squares discriminant analysis (OPLS-DA) was employed to investigate the types and content of endogenous peptides to screen out potential biomarkers that could indicate pork spoilage during storage. This study provides an alternative method with potential to improve the meat-freshness evaluation system, thereby improving meat safety during retail and storage.

## 2. Materials and Methods

### 2.1. Materials and Sample Preparation

A total of 40 *longissimus lumborum* muscles (after 24 h postmortem, from 6 months old crossbred, castrated male pigs (Duroc × Landrace × Large Yorkshire), which were economically fed and slaughtered in a slaughtering workshop at 10–15 °C; 3 h after slaughter, carcasses were chilled at 4 °C, and bone out at 24 h) were sampled at Beijing Hualian Group, Nanjing, China. All samples were transported via insulated chilled boxes to the laboratory within 2 h and aseptically prepared to remove the fat and connective tissues. Subsequently, all *longissimus lumborum* muscles were cut into 5 × 7 × 3 cm chunks of a similar size (approximately 60 g) on a clean bench and randomly assigned into four groups representing storage conditions of −2, 4, 10, and 25 °C. They were placed onto a plastic tray (23 × 14 × 7 cm, Cryovac TQBC-1175, Sealed Air Co., Ltd., Shanghai, China), sprayed with 95% ethyl alcohol, and then wrapped with two layers of commercial polyethylene (PE) cling wrap (Miaojie, TOP Group, Shanghai, China). For the 25 °C group, five time points (0, 5, 10, 15, and 20 h) and 45 pieces (nine individual pieces per time point) were utilized for analysis. Consistent with the 25 °C group, six time points (0, 5, 10, 15, 20, and 25 days) and 54 pieces; six time points (0, 3, 6, 9, 12, and 15 days) and 54 pieces; and seven time points (0, 12, 24, 36, 48, 60, and 72 h) and 63 pieces were used for the analyses of the −2, 4, and 10 °C groups, respectively. Four individual pieces were randomly selected for the measurements of basic indicators (color, pH, TVC, and TVB-N). Five individual pieces per time point were randomly selected for peptidomics analysis.

A new batch of pork samples was sampled from three markets in Nanjing (Beijing Hualian Group, Weigang Market, and Suguo Supermarket, Nanjing, China) with 54 *longissimus lumborum* muscles (18 from each market). All samples were transported via insulated chilled boxes to the laboratory within 2 h and aseptically prepared to remove the fat and connective tissues. Each muscle in each supermarket was cut into 3 pieces (the same treatment as mentioned above). For each temperature group (4 and 25 °C), 27 pieces (nine pieces per time point) collected at three time points (fresh, accelerated spoiled, and spoiled states) were utilized for analysis.

### 2.2. Color Measurement

The method described by Hunt et al. [21] was adopted to measure sample color. Before measurement, the colorimeter (CR-40, Minolta Camera Co., Osaka, Japan) was calibrated with a white porcelain plate (mod CR-A43). After samples bloomed at 4 °C for 30 min, the lightness (L*), redness (a*), and yellowness (b*) of pork surfaces were measured with illuminant D 65, viewing angle 0°, and viewing area diameter 8 mm at three different locations of samples exposed to the air. All measurements were made in quadruplicate (*n* = 4).

### 2.3. Microbiological Analysis

The TVC was determined following the method by Ye et al. [22]. Briefly, 25 g from each group per sampling time was transferred aseptically into a sterile stomacher bag containing 225 mL of sterile saline and then homogenized for 2 min using a stomacher machine (BagMixer 8400 VW, Interscience Co., Bretesche, France). After serial dilution, 1 mL of each sample was inoculated on plate count agar (Luqiao Co., Ltd., Beijing, China) and incubated at 37 °C for 48 h. The counts, measured in quadruplicate (*n* = 4), were expressed as log colony-forming units per gram (log CFU/g).

### 2.4. pH

pH was measured as described by Lan et al. [23] using a Hanna 211 pH meter (Hanna, Villafranca Padovana, Italy) calibrated using pH buffers (4.0, 7.0, and 10.0) for precision. Briefly, 1 g of meat sample was homogenized with 10 mL of ice-cold buffer containing sodium iodoacetate (5 mM) and potassium chloride (150 mM) at 6000 rpm for 1 min (10 s interval) in an ice-bath. Each measurement was performed in quadruplicate (*n* = 4).

### 2.5. Total Volatile Basic Nitrogen

The TVB-N content in samples was determined as described by Bassey et al. [24]. Briefly, 10 g sample per group per sampling time was homogenized (Ultra-Turrax T25, IKA, Berlin, Germany) in 100 mL distilled water and stirred for 30 min. Then, 10 mL of the filtrate was mixed with 1 g MgO and analyzed using an automatic Kjeldahl nitrogen analyzer (Kjeltec 2300, Foss Company, Hillerod, Denmark). The results, measured in quadruplicate, were expressed as milligram of N per 100 g of pork meat.

### 2.6. Sample Preparation for Peptidomics

Peptide extraction was performed as described by Azkargorta et al. [25], with minor changes. Meat samples (0.1 g) collected in the fresh, accelerated spoiled, and spoiled states (five samples per state) at each temperature group were homogenized (8500 rpm, 5 × 20 s) with 0.2% formic acid (FA; ROE, Newark, New Castle, DE, USA) containing 0.2% acetonitrile (ACN; Merck, Pittsburgh, PA, USA) to precipitate high-abundance proteins. After centrifugation (12,000× *g*, 30 min, 4 °C), 300 μL of supernatant was diluted with 900 μL ACN. The mixture was incubated for a further 30 min at 4 °C, followed by centrifugation (12,000× *g*, 10 min). The resulting supernatant was evaporated and re-dissolved in 0.2% FA containing 0.2% ACN and then centrifuged (12,000× *g*, 10 min, 4 °C). Thereafter, 2 and 5 μL of each sample solution were used for peptide and quality control (QC) analyses, respectively.

### 2.7. Peptide Identification and Data Analysis

A UHPLC-LTQ-Orbitrap mass spectrometer (Thermo Fisher Scientific, Waltham, MA, USA) was used to determine the untargeted peptide profiles, as described by Miao et al. and Stefan et al. [26,27], with minor modifications. Gradient elution was conducted between 100% ACN (A) and water (B) containing 0.1% (*v*/*v*) FA as follows: 0–5 min, 3–3% B; 5–45 min, 3–35% B; 45–46 min, 35–95% B; 46–56 min, 95–95% B; 56–57 min, 95–3% B; and 57–63 min, 3–3% B. The flow rate was 0.2 mL/min. The temperature of the C18 column (250 mm × 3 mm × 5 μm) was maintained at 35 °C. The column outlet was directly coupled to electron spray ionization (ESI) with an ionization voltage set at 3 kV for the positive scan. Nitrogen was used as the sheath (35 arb) and aux (10 arb) gas. The scanning range of the primary mass spectrum was 200–1200 *m*/*z*, and the resolution was 60,000. A data-dependent acquisition (DDA) scan was conducted for collision-induced dissociation (CID) model. The 10 ions with the highest intensity were selected for secondary fragmentation analysis with a collision energy of 35 eV.

The acquired raw data were imported into PEAKS software (Bioinformatics Solutions, Waterloo, Canada) for peptide identification and quantification. The parameters were as follows: oxidation of methionine as variable modification, parent mass error tolerance (15.0 ppm), fragment mass error tolerance (0.6 Da), database (UniProt *Sus scrofa*), and filter charge (1–3). The peak area of each identified peptide was integrated using the PEAKS Q module and subjected to logarithmic (log) conversion. The normalized peak area of each peptide after log_2_ conversion was imported into SIMCA-P 14.0 software (Umetrics, Umea, Sweden) for multivariate analysis. To identify potential markers, variable importance projection parameter value > 1, fold-change > 1.3 or <0.77, and *p* < 0.05 [28], were used as the screening criterion to compare the OPLS-DA data in a pairwise manner. The screened out peptide sequence was then submitted to Genscript (Nanjing, China) for synthesis.

### 2.8. Quantification of Potential Peptide

A total of 5 μL of each sample treated as detailed above was injected. LC analysis was performed using a UHPLC system (DIONEX, Thermo Scientific, Sunnyvale, CA, USA) equipped with an auto-sampler, a vacuum degasser unit, a quaternary pump, and a column compartment. Samples were separated using a Gemini^®^ 5 μm NX-C18 Column (250 × 3 mm, 110 Å, Phenomenex, Torrance, CA, USA) at 35 °C. The mobile phases consisted of acetonitrile (A) and water containing 0.1% FA (B), and the elution gradient was set as follows: 3% A (0–0.5 min), 3–35% A (0.5–8.5 min), 35–95% A (8.5–9 min), 95% A (9–14 min), 95–3% A (14–14.1 min), and 3% A (14.1–20 min). The flow rate was fixed at 0.2 mL/min. MS analysis was performed using an LTQ-Orbitrap XL mass spectrometer (Thermo Electron, San Jose, CA, USA) equipped with a heated electrospray ionization interface. The ionization voltage was set at 3 kV for the positive scan. The capillary temperature was fixed at 300 °C. Nitrogen served as both the sheath (35 arb) and auxiliary (10 arb) gas. From 0 to 20 min, the results of PEAKS software were imported into Skyline 3.0 software (MacCoss, Washington, USA) to select the parent-fragment ion pairs of the potential peptide. The precursor ion was selected using CID model with normalized collision energy of 35%.

### 2.9. Statistical Analysis

The results of color, microbiological analysis, pH, and TVB-N were analyzed by one-way analysis of variance (ANOVA) using SPSS for Windows version 20 (SPSS Inc., Chicago, IL, USA). Duncan’s multiple range test was used to compare significant differences at *p* < 0.05. The results of color, microbiological analysis, pH, and TVB-N are expressed as the mean and standard deviation of four replicates. The peptide MVHMASKE content was analyzed by two-way analysis of variance (ANOVA) with Bonferroni post hoc test, and the results are expressed as the mean and standard deviation of nine replicates.

Peptide sequencing and quantification were performed by PEAKS software (Bioinformatics Solutions, Waterloo, Canada). Principal component analysis (PCA) and orthogonal partial least-squares-discriminant analysis (OPLS-DA) were analyzed with SIMCA-P 14.0 software (Umetrics, Umea, Sweden) for overall variable analysis. Pearson correlation coefficients were used to assess the relationship between intensity of peptide MVHMASKE and changes in basic meat-freshness indicators. Results for the selected biomarkers were output and analyzed by Skyline 3.0 software (MacCoss, Washington, DC, USA).

## 3. Results and Discussion

### 3.1. Changes in Color

**Figure 1:**
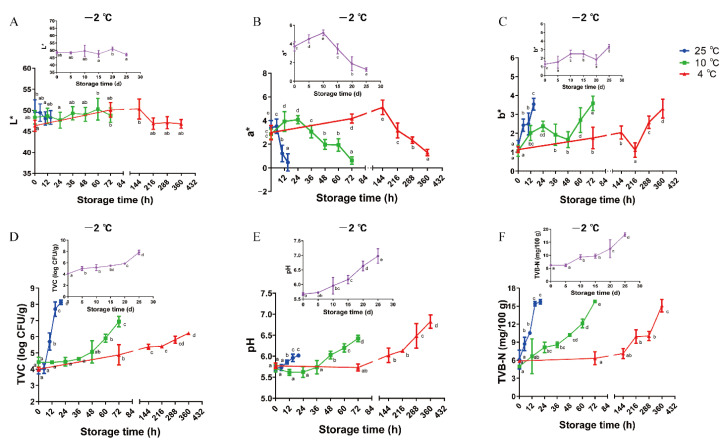
Changes in L* (**A**), a* (**B**), b* (**C**), TVC (**D**), pH (**E**), and TVB-N (**F**) in pork under storage at −2, 4, 10, and 25 °C. Error bars indicate the standard deviation of four repeated experiments. The different letters (a–e) indicate significant differences between different storage times under the same temperature (*p* < 0.05).

A bright red color was used to denote meat freshness. Except for lightness (L*), redness (a*) and yellowness (b*) were markedly affected by storage temperature and sampling time (Figure 1A–C). Although no significant variations were observed in L* values (*p* > 0.05), the a* values slightly increased at the beginning of storage and then declined over time, while b* values trended upwards throughout storage period. Compared with low-temperature (−2 and 4 °C) storage samples, the a* values decreased rapidly after 24 and 5 h in samples stored at 10 and 25 °C, respectively (Figure 1B). According to Lv et al. [29], a* decline can be induced by oxidation and formation of metmyoglobin. Conversely, significant variations (*p* < 0.05) in b* values were observed from 20 d, 9 d, 48 h, and 10 h in the −2, 4, 10, and 25 °C storage groups, respectively. An increase in b* content is associated with yellow pigment derived from the reaction between lipids and the amines in protein amines or phospholipid head groups [30].

### 3.2. Microbiological Analysis

Bacterial growth induces the deterioration of meat freshness during storage [31]. The TVC was determined to examine the impact of spoilage bacteria in pork samples during storage (Figure 1D). The initial TVC values (−2 °C = 4.06, 4 °C = 3.98, 10 °C = 4.44, and 25 °C = 3.92 log CFU/g) were similar with the count (4.49 log CFU/g) reported by Zhou et al. [32] in chilled pork and that (3.50–4.00 log CFU/g) reported by Ye et al. [22] and Ding et al. [33] in super-chilled pork. An upward trend was observed throughout storage period. The TVC content in the 4, 10, and 25 °C groups rapidly exceeded the permissible threshold of 6 log CFU/g established by China National Food Safety Standard (GB/T9959.2-2008) at 15 days (6.22 log CFU/g), 72 h (6.94 log CFU/g), and 15 h (7.70 log CFU/g), respectively, while the −2 °C group did not exceed the threshold until 25 days. This indicates that high temperature contributes to the rapid proliferation of microorganisms. The disparity in counts could arise from the storage conditions, as the microbiota in samples, except under storage at −2 °C, contained different mesophilic and psychotropic species [11]. This corroborates the report of Ye et al. [22], who stated that temperatures lower than 0 °C could effectively control the microbiological activities in meat.

### 3.3. Changes in pH

pH is associated with meat freshness, as it reflects the degree of protein degradation and accumulation of spoilage metabolites [34]. A previous study reported that meat begins to deteriorate at around pH 6.3–6.5 [35]. Despite significant variation (*p* < 0.05) in each group during the storage period (Figure 1E), the 10 and 25 °C groups presented a similar trend and increased at 36 and 10 h (*p* < 0.05), with the results ranging from 5.70 to 6.39 and from 5.71 to 6.00, respectively. Conversely, the values obtained for the −2 and 4 °C groups significantly increased (*p* < 0.05) after 10 days and 6 days, respectively. Similarly, an upward trend has been observed for rabbit meat stored at 4 °C [23]. The slight decline in pH may be attributed to the rapid consumption of adenosine triphosphate, inducing the accumulation of inorganic phosphate [23] and lactic acid produced through glycogen decomposition [36]. Conversely, an increase in pH is due to the hydrolysis of meat protein by endogenous enzymes [4] or the production of nitrogenized basic compounds by meat-spoilage microorganisms [34].

### 3.4. Total Volatile Basic Nitrogen Analysis

The TVB-N results showed a progressive trend in all groups, with substantially higher values under high-temperature (10 and 25 °C) groups compared with low-temperature (−2 and 4 °C) groups (Figure 1F). Throughout the storage period, the TVB-N values increased rapidly, ranging from 4.97 to 15.78 mg/100 g and 6.10 to 15.74 mg/100 g in 10 and 25 °C groups, respectively, exceeding the acceptable limit of 15 mg/100 g for fresh pork according to China’s standard protocol (GB/T 2707-2005). While TVB-N contents in the −2 and 4 °C groups increased slowly during the initial storage period, a marked increase was observed after 15 days and 6 days, and exceeding values were observed at 25 days and 15 days, respectively. This result was consistent with the findings of Bassey et al. [24,37], who demonstrated that TVB-N exceeded 16 mg N/100 g and 19 mg N/100 g in air-packed pork stored at −2 and 4 °C, respectively. In addition, Sun et al. [4] reported that TVB-N values of yak meat stored at −2 °C exceeded 15 mg/100 g after 24 days. Liu et al. [38] reported a rapid increase in TVB-N after 14 days, with the values for meat stored at −3 °C being markedly lower than those of meat stored at −1 °C. Several factors, such as pH increase during postmortem storage [39], protein degradation reactions initiated by spoilage bacteria and endogenous enzymes [4], including the oxidation of amines, degradation of oxides, and the deamination and decarboxylation of free amino acids, may induce an increase in TVB-N [24].

Considering basic indicators, the period of accelerated spoilage ranges from 15 days to 25 days, 9 days to 15 days, 36 h to 72 h, and 10 h to 15 h in the −2, 4, 10, and 25 °C groups, respectively. The results obtained were consistent with the findings of Tang et al. [1], who reported that the shelf-life of pork stored at 10 °C was 3 days, and Ding et al. [33], who reported that shelf-life of pork stored at −2 °C was 25 days. Thus, samples at the fresh, accelerated spoiled, and spoiled states were collected and used to mine potential peptide biomarkers for the evaluation of pork freshness.

### 3.5. Identification of Peptides

Non-targeted peptidomics was applied to assess changes of peptide profile during pork-storage period. To ensure high reliability and repeatability, the FDR value of the database search algorithm was set to 1%, and the variation coefficient of the peptide response area presented in the QC was <15%. Based on this criterion, 79, 215, 194, and 337 peptides were identified from the −2, 4, 10, and 25 °C groups, respectively (Supplementary Table S1). Herein, precursor proteins identified from the samples stored under 10 and 25 °C represented more categories than those of samples stored under −2 and 4 °C and mainly included metabolic enzymes, structural proteins, and transport proteins (Figure S1). Generally, a peptide/protein with molecular weight > 5 kDa undergoes inefficient ionization in the ESI source [40], indicating marked degradation in the 10 and 25 °C groups. Lan et al. [23] also observed a lower degree of protein decomposition in rabbit muscles stored at −4 °C when compared to those stored at 4 °C. An increase in temperature accelerates microbial growth, which results in rapid protein degradation [3,11]. Of note, more peptides were identified in 25 °C storage group. We speculate that except the effect of microbial growth, several endogenous proteolytic systems presented in meat, including calpains, proteasomes, cathepsins, and other serine peptidases, may also play an intense role. Endogenous enzymes are sensitive to temperature, especially the calpain system, which is responsible for the majority of postmortem degradation of structural proteins, such as myofibrillar proteins, including myosin, tropomyosin, desmin, titin, and nebulin [41]. Additionally, previous study reported that the activity of calpains is significantly higher under elevated temperatures (25 °C) compared to 0 °C [42].

### 3.6. Multivariate Analyses of Pork Peptidome

**Figure 2:**
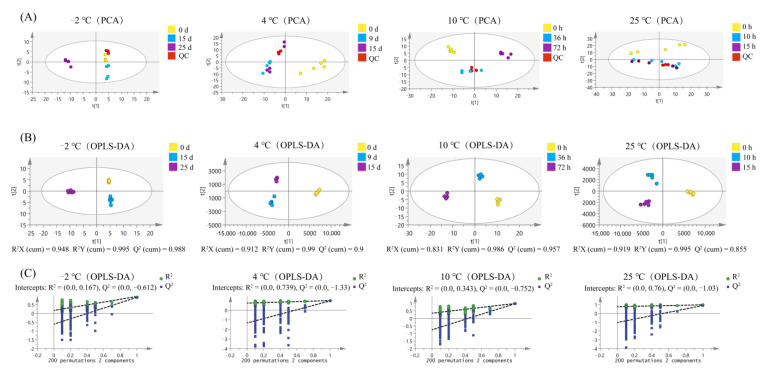
PCA (**A**), OPLS-DA (**B**), and permutation test (**C**) of pork meat identified peptides at different time points under storage at −2, 4, 10, and 25 °C.

SIMCA-P software was used to perform multivariate analyses on the peptide intensity for different pork states. The PCA score plots (Figure 2A) indicated that all samples were within the 95% confidence ellipse of Hotelling’s *T*^2^ test, and the aggregation degree of QC (red dots in Figure 2A) reflected the reliable repeatability of peptidomics in the present study. The PCA illustrated that clustering patterns varied between different groups. For instance, samples from 25 days in the −2 °C storage group were distinct from others in the *t* (1) axis, while there were obvious separations on day 0, day 9, and day 15 in the 4 °C storage group. In the 10 °C storage group, samples collected from different time points were significantly distinguished from each other. In contrast, samples collected at 0 h in the 25 °C storage group were separated from samples collected at other time points on the *t* (2) axis. However, the distinction between samples at certain time points remained ambiguous, suggesting the need for further data analysis.

To establish a clear distinction in groups between different sampling times, OPLS-DA, a supervised chemometrics approach, was performed to sensitively separate the samples and provide an accurate prediction (Figure 2B). Model parameters of *R*^2^*X* (cum), *R*^2^*Y* (cum), and *Q*^2^ (cum) revealed satisfactory fitness and predictability of the model. To evaluate the efficacy of the OPLS-DA model, 200 permutation tests were performed (Figure 2C). Both *R*^2^ and *Q*^2^ were lower than the original value of the model, demonstrating that the model was not overfitting. Cluster analysis (Figure 2B) revealed that the peptidome of the samples differed between storage states in each group during storage period, indicating that meaningful data exploration for differentially abundant peptides (DAPs) can be performed.

### 3.7. Selection of Potential Peptide Biomarkers

To find the potential peptide biomarkers that could characterize pork states during storage period, a volcano plot was created to compare two different meat states: (1) fresh to accelerated spoiled (SS1) and (2) accelerated spoiled to spoiled (SS2) states (Figure S2). Among these identified peptides, 20, 13, 16, and 8 were upregulated, and 6, 36, 37, and 50 were downregulated for SS1 in the −2, 4, 10, and 25 °C groups, respectively. In addition, 15, 28, 16, and 12 upregulated and 22, 60, 32, and 47 downregulated peptides were detected in SS2, respectively. Since there could be repeated peptides with the same changes in both states, non-repetitive upregulated (27, 36, 22, and 17) and downregulated (27, 38, 27, and 40) peptides were obtained as DAPs in −2, 4, 10, and 25 °C groups, respectively.

**Figure 3:**
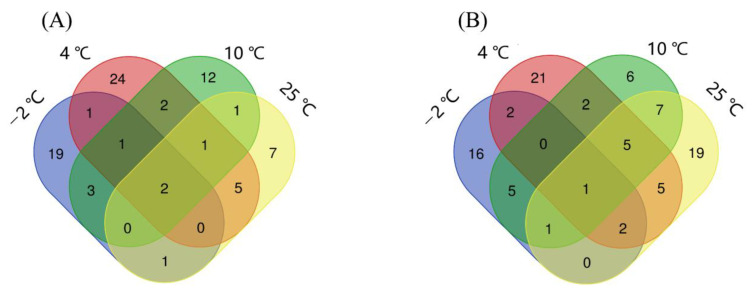
Venn diagrams of differentially abundant peptides. Upregulated peptides (**A**); downregulated peptides (**B**).

A Venn diagram was constructed to identify the peptides presented in each group (Figure 3). Three peptides (MVHMASKE, APPPPAEVHEVH, and PPPAEVHEVH) identified in all groups were screened out, and they were either significantly upregulated or significantly downregulated in one of the comparisons (SS1 or SS2) under each temperature. However, for an accurate indication of pork meat freshness, a peptide that exhibited the same continuous trend in all groups was selected as a potential marker. Thus, combined with the volcano plot result, MVHMASKE was identified as the only peptide showcasing a distinct downward trend and was therefore considered to be a potential marker. The precursor protein of peptide MVHMASKE was glyceraldehyde-3-phosphate dehydrogenase, which is a key enzyme involved in the catalysis of glycolysis and regulates NADH production [43]. During storage, NADH decreased with the decline of glyceraldehyde-3-phosphate dehydrogenase activity and thereby could reduce the metmyoglobin-reducing activity (MRA) and color stability of pork meat [43].

**Figure 4:**
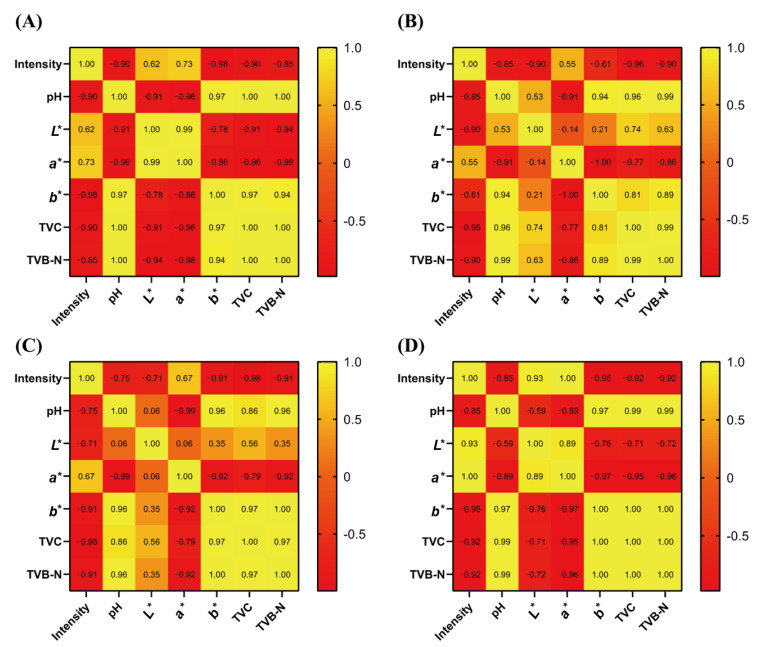
Pearson correlation between intensity of peptide MVHMASKE and basic freshness indicators in pork under storage at −2 °C (**A**), 4 °C (**B**), 10 °C (**C**), and 25 °C (**D**).

Although no studies have investigated the use of peptides to indicate pork meat freshness, Chen et al. [20] identified four endogenous peptide markers as potential indicators to evaluate the shelf-life of *C. gigas* during anhydrous storage at 4 °C. However, the relationship between peptide content and shelf-life of *C. gigas* was not determined. To ascertain the relationship between peptide MVHMASKE and meat freshness, Pearson correlation (Figure 4) was calculated between the intensity of peptide MVHMASKE and changes in basic pork indicators. The result revealed a negative correlation between intensity and TVC/TVB-N values in each storage temperature, suggesting the possibility of using MVHMASKE as a marker for evaluating pork-meat freshness.

### 3.8. Verification of Potential Peptide Markers by Parallel Reaction Monitoring (PRM) Determination and Real-Life Sample Analysis

PRM, a targeted method for quantifying selective proteins/peptides, provides high sensitivity and precision, with easier data acquisition than multiple reaction monitoring (MRM) [44]. To perform PRM, skyline software was used to select the parent-fragment ion pairs of peptide MVHMASKE (precursor ion 466.7206 (2^+^); the two most intense fragment ions: 351.81 (y6^2+^) and 565.30 (y5^+^)) (Figure S3). Then, the effectiveness of the potential peptide biomarker in evaluating the sample freshness was verified. A new batch of pork samples was obtained from three markets in Nanjing (Beijing Hualian Group, Weigang Market, and Suguo Supermarket). The samples were pretreated in the same way as described above and stored at 4 and 25 °C for verification. Three samples were collected from each time point to analyze the content changes of peptide MVHMASKE.

**Figure 5:**
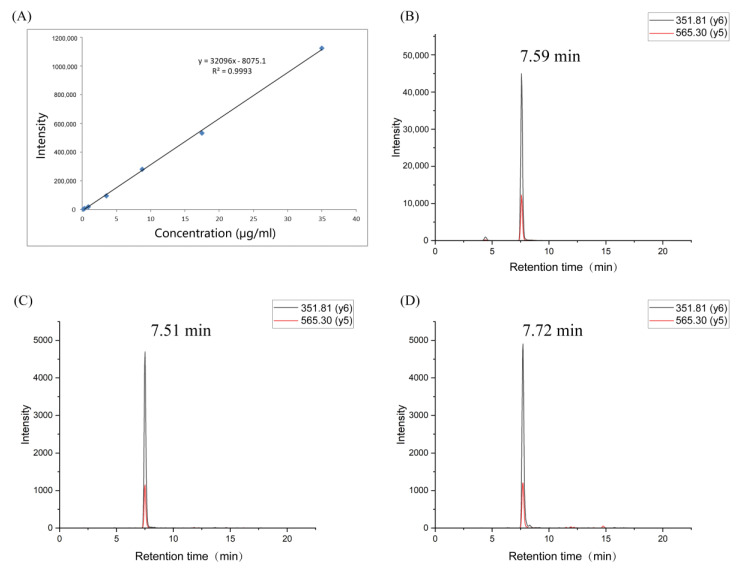
Scatter plot between the standard concentration and peak area of peptide MVHMASKE (**A**). Representative extracted ion chromatogram of synthesized peptide MVHMASKE (**B**) and identified peptide MVHMASKE in samples during storage at 4 °C (**C**) and 25 °C (**D**).

**Figure 6:**
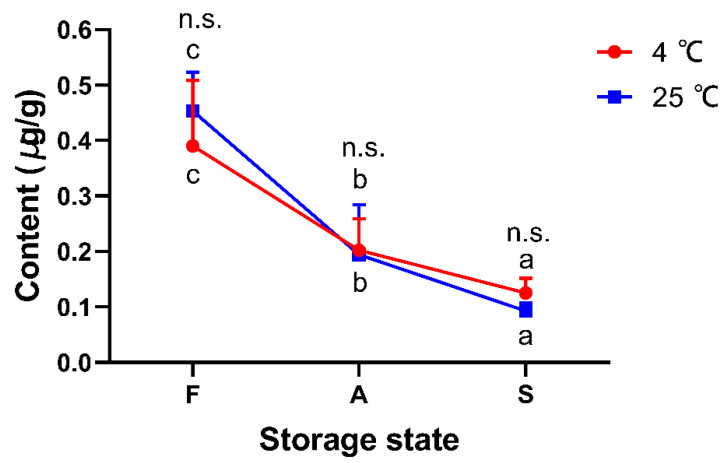
Changes in the content of peptide MVHMASKE in pork sampled from three markets in Nanjing under storage at 4 °C and 25 °C. F, fresh state; A, accelerated spoiled state; S, spoiled state. The different letters (a–c) indicate significant differences between different storage states at the same temperature (*p* < 0.05). n.s. indicates no significant differences between two temperatures at the same storage state (*p* > 0.05).

**Figure 7:**
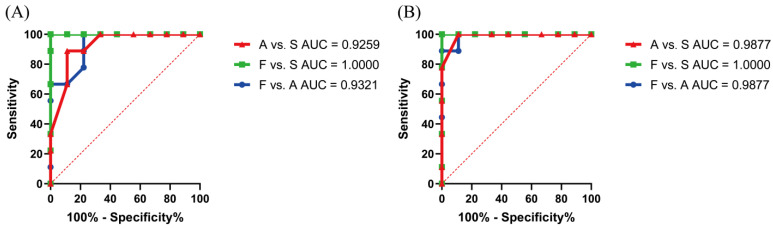
The ROC curve of peptide MVHMASKE in pork under storage at 4 °C (**A**) and 25 °C (**B**). F, fresh state; A, accelerated spoiled state; S, spoiled state.

According to the results of the representative extracted ion chromatogram of peptide MVHMASKE from pork meat and the synthesized peptide, the peak shape was sharp with little interference and coincided well. The retention time for peptide MVHMASKE from pork meat was consistent to that of the synthesized peptide, being within ±2% [20], illustrating the amino acid sequence was confirmed (Figure 5B–D). An external standard quantitative method was used to analyze the synthesized peptide and the target peptide extracted from the samples. There was a strong linear relationship (R^2^ = 0.9993) between the concentration of standard peptide (0.175 to 35 μg/mL) and the peak area of peptide MVHMASKE (Figure 5A), which could quantify the peptide. Subsequently, the contents of peptide MVHMASKE were calculated, and the results are presented in Figure 6. The MVHMASKE content in all samples showed an obvious decreasing trend during the storage period, indicating the changes of pork from fresh to spoiled. A receiver-operating characteristic (ROC) curve was performed to evaluate whether the potential peptide can be used as a freshness biomarker. The area under the ROC curve (AUC) ranging from 0.9 to 1.0 indicated that the predictive power of the variate was good [45]. An excellent differentiation (all AUCs > 0.9) among different states of pork meat under each temperature (Figure 7) was observed, which indicated good discriminatory sensitivity. Notably, although only one unique peptide was identified in the present study, this is an explorative study to identify a peptide as a biomarker for characterizing pork-meat freshness under multi-temperature storage. The results also demonstrated the key proteins that were degraded after pork spoilage under different storage conditions and revealed pork spoilage at the peptide level. Combined with these results, our study demonstrated that monitoring the change of peptide MVHMASKE may be applied as a way to support evaluating pork meat freshness.

## 4. Conclusions

In this study, basic freshness indicators were monitored to evaluate the pork-meat freshness under different storage temperatures. Our results revealed variations in the spoilage processes of pork meat stored at different temperatures. Hence, by integrating all parameters, time points of fresh, accelerated spoiled, and spoiled states of the samples under different conditions were ascertained. A peptide biomarker MVHMASKE was screened out via multivariate analyses, and the PRM coupled with the external standard quantitative method was established to analyze changes in peptide content using real-life samples. The potential of peptide MVHMASKE was verified; however, the thresholds of this peptide to well define the freshness change of pork meat should be established with a larger size of testing samples and more accurate quantitative analysis in further study. In addition, as the first step, only one peptide was considered as common potential indicator of meat spoilage under all four storage temperatures. To elucidate this matter further, subsequent work needs to be done as a combined strategy to explore more peptides as potential biomarkers at each specific temperature. Overall, this peptidomics-based method for mining biomarkers is a prospective alternative to evaluate pork meat freshness, and the result of this study provides the new theoretical basis for the standardized system in assessing pork quality.

## Data Availability

The peptidomics datasets generated for this study can be found in ProteomeXchange, Accession No. PXD031995.

## Supplementary Materials

The following supporting information can be downloaded at: https://www.mdpi.com/article/10.3390/foods11081144/s1, Figure S1: The proportion of mapped precursor proteins from identified peptides in pork under storage at −2 °C (A), 4 °C (B), 10 °C (C), and 25 °C (D); Figure S2: Volcano plot of differentially abundant peptides identified at different time points under storage at −2 °C (A), 4 °C (B), 10 °C (C), and 25 °C (D); Figure S3: MS/MS spectra of peptide MVHMASKE obtained by UHPLC-LC-LTQ Orbitrap XL; Table S1: Detailed information on the identified peptides in pork under storage at −2, 4, 10, and 25 °C.
