# Supplementary figures and images for: Identification of Potential Peptide Marker(s) for Evaluating Pork Meat Freshness via Mass Spectrometry-Based Peptidomics during Storage under Different Temperatures
Source: Foods. 2022 Apr 15;11(8):1144. doi: 10.3390/foods11081144 (PMC9027284; doi:10.3390/foods11081144)

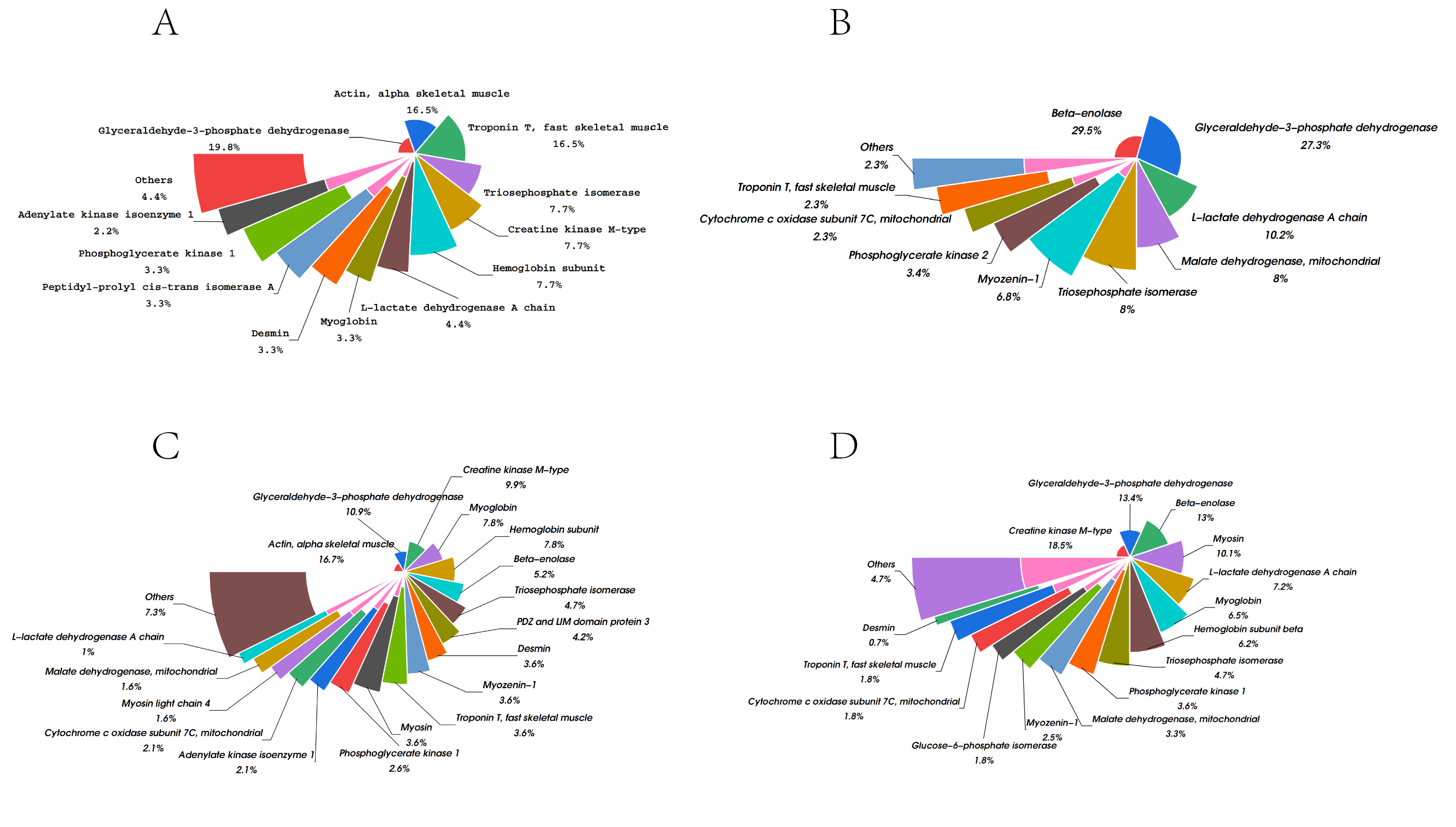

Supplement: Supplementary file 1 [file foods-11-01144-s001.zip › Fig.S1.tif]

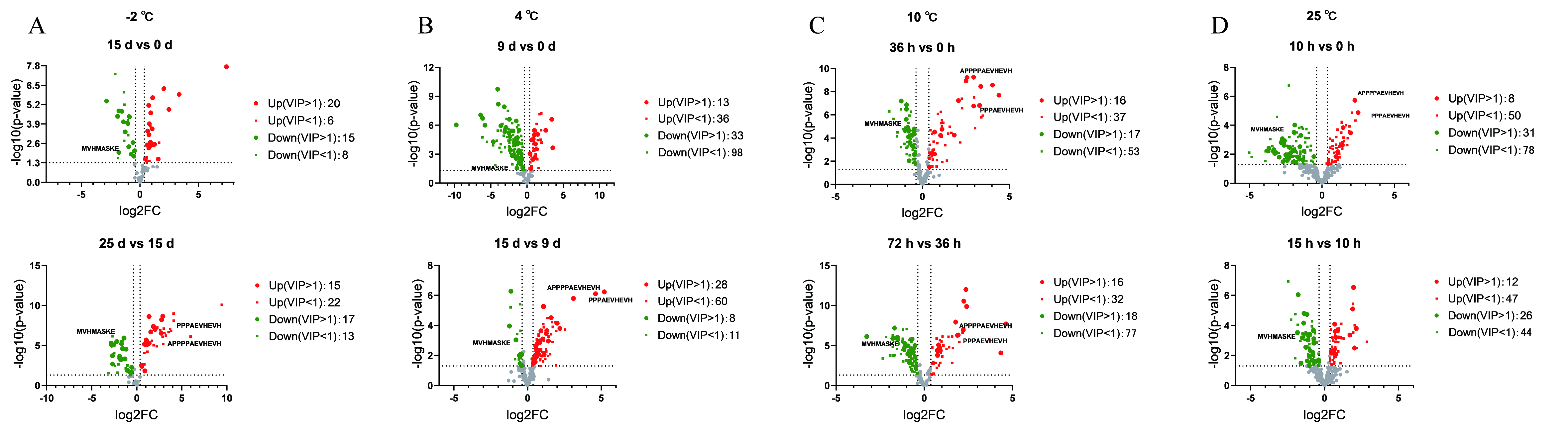

Supplement: Supplementary file 1 [file foods-11-01144-s001.zip › Fig.S2.tif]

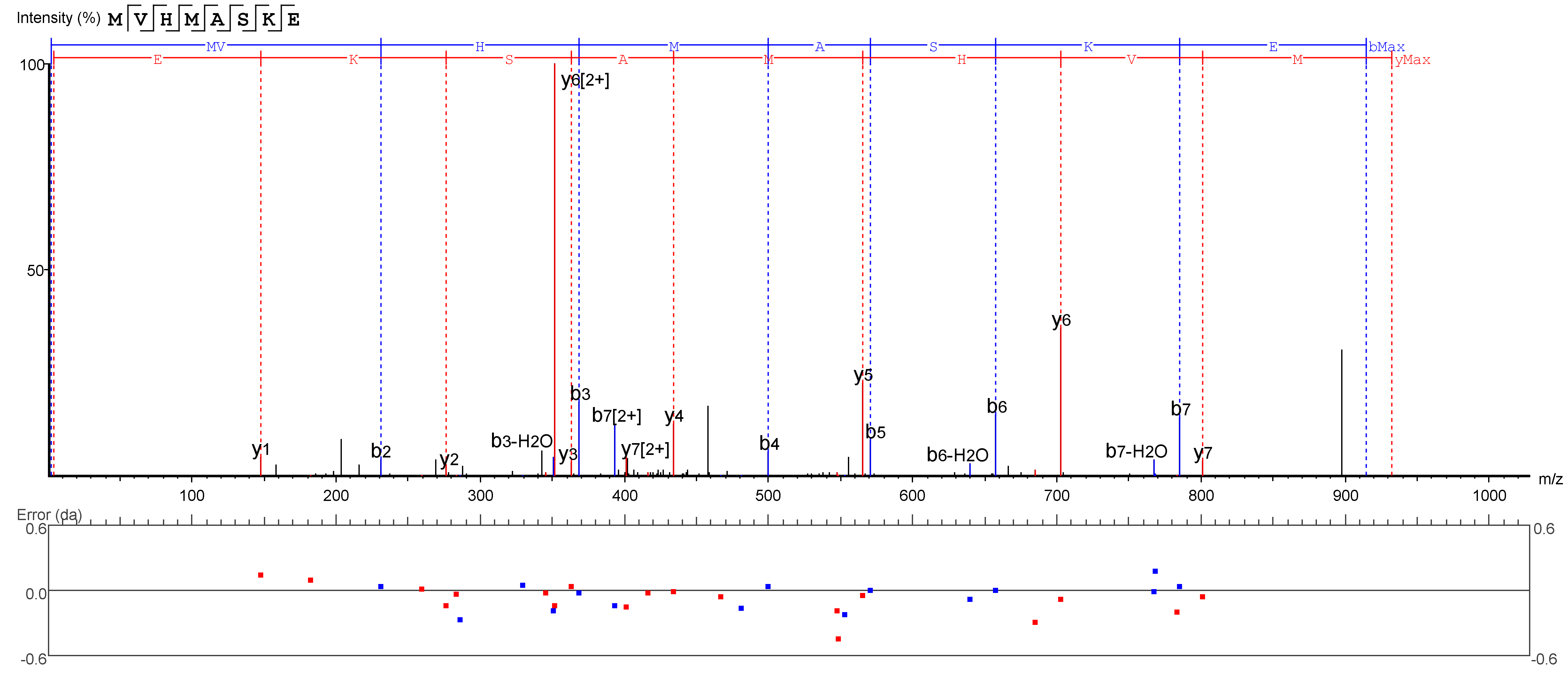

Supplement: Supplementary file 1 [file foods-11-01144-s001.zip › Fig.S3.tif]
